# Editorial: Genetic and Epigenetic Basis of Neurodegenerative Diseases

**DOI:** 10.3389/fnagi.2022.958847

**Published:** 2022-06-27

**Authors:** Lamei Yuan, Yi Guo, Shu Wen, Hao Deng

**Affiliations:** ^1^Health Management Center, The Third Xiangya Hospital, Central South University, Changsha, China; ^2^Center for Experimental Medicine, The Third Xiangya Hospital, Central South University, Changsha, China; ^3^Disease Genome Research Center, Central South University, Changsha, China; ^4^Department of Neurology, The Third Xiangya Hospital, Central South University, Changsha, China; ^5^Department of Medical Information, School of Life Sciences, Central South University, Changsha, China; ^6^Department of Molecular and Human Genetics, Baylor College of Medicine, Houston, TX, United States

**Keywords:** variants, epigenetic modifications, neurodegenerative disease, degeneration, neuronal cell death

Neurodegenerative diseases, such as Alzheimer's disease (AD), Parkinson's disease (PD), Huntington's disease (HD), and amyotrophic lateral sclerosis (ALS), are characterized by progressive functional degeneration and ultimate neuronal cell death, causing problems with motor and/or cognitive functions and further seriously affecting patients' activities of everyday living (Shany-Ur et al., 2014; Deng et al., 2018; Katsuno et al., 2018; Andreone et al., 2020). The debilitating and incurable neurodegenerative diseases pose a threat to the health of millions of people worldwide, accounting for a big chunk of the global disease burden (Slanzi et al., 2020). As the population is aging, the negative impacts of neurodegenerative diseases on patients and society will be worse (Maher, 2021).

The complex combination of pathogenic variants, epigenetic modifications, and environmental factors contributes to the risk of neurodegenerative diseases. Genetic factors play major roles in the pathogenesis of neurodegeneration as monogenic causes for Mendelian disorders or susceptibility modifiers for sporadic complex diseases (Pihlstrøm et al., 2017; Andreone et al., 2020; Ghosh and Saadat, 2021). Additionally, copy number variants caused by duplication, insertion, or deletion of DNA segments usually larger than 50 base pairs, and epigenetics, which builds a bridge between the environmental information and gene expression through epigenetic regulations such as DNA methylation, RNA-mediated mechanisms, and histone protein modifications, also lead to disease phenotypes (Jirtle and Skinner, 2007; Ghosh and Saadat, 2021; Coutelier et al., 2022). The widespread applications of high-throughput technologies in biology and medicine, such as next-generation sequencing and DNA microarray (gene chip) technology, are generating an increasing body of evidence which broadens the understanding for genetic and epigenetic basis of neurodegenerative diseases.

This topic made efforts to recruit high-quality articles covering genetic and epigenetic relevant factors and their pathogenic mechanisms involving in neurodegenerative diseases. These researches will ultimately advance evolvements of genetic diagnosis approaches, better care to patients especially in advanced age, personalized therapeutic strategies, and prevention of recurrence in families. After rigorous peer review, we gathered six articles described as follows.

Chronologically, in the first article “Identification of Genetic Networks Reveals Complex Associations and Risk Trajectory Linking Mild Cognitive Impairment to Alzheimer's Disease”, Strafella et al. used approaches of single nucleotide polymorphism (SNP) genotyping followed by statistical, bioinformatic and machine-learning analyses to explore the associations of gene and miRNA variants with amnestic mild cognitive impairment (aMCI) and sporadic AD. The study identified 21 aMCI and 13 sporadic AD associated SNPs, and a set of variants shared between aMCI and AD. The higher odd ratios of shared variants in AD patients revealed more susceptibility of developing AD in aMCI patients with these shared variants. Furthermore, among the related genes and miRNAs, some were found to involve in neuroinflammation and neurodegeneration underlying aMCI and sporadic AD.

In the article “Emerging Potential of Exosomal Non-coding RNA in Parkinson's Disease: A Review”, Zhang et al. firstly systematically summarized the neuroprotection of exosomal non-coding RNA (ncRNA) including maintaining synaptic function, neuromuscular junction, neurodevelopment, and neuroimmune function. The potential biomarkers of exosomal ncRNA in PD were listed to provide a reference on helping early diagnosis. The authors emphasized their potential roles in PD progression when dysregulations occur through mechanisms of α-synuclein aggregation, activation of autophagy and inflammasome, oxidative stress, and dopamine neuron death. The PD treatment strategies and tailored medicines based on exosomal ncRNA were also mentioned.

Subsequently, the third article, “The Emerging Roles of the β-Secretase BACE1 and the Long Non-coding RNA BACE1-AS in Human Diseases: A Focus on Neurodegenerative Diseases and Cancer” by Sayad et al., overviewed that β-secretase (BACE1) and its antisense transcript, a positive regulator, BACE1-AS, play roles in various human diseases, particularly amyloid-linked neurodegenerative diseases, such as AD and PD. Both BACE1 and BACE1-AS suggest potentials on diagnosis as biomarkers of a simple, reliable, and minimally invasive detection method, but also on targeted therapy to diminish amyloidogenesis.

The fourth article was “Discovery of Novel Drug Candidates for Alzheimer's Disease by Molecular Network Modeling” by Zhou et al. AD-associated genes from three datasets were found to be enriched in two modules (M3 and M8) of gene co-expression networks. The dysregulation of M3 and M8 modules in AD was then validated by differential expression analysis. Through targeting the underlying dysregulated gene network, Gly-His-Lys acetate salt was proposed as a candidate of anti-AD drugs by utilizing the Connectivity Map database, and its neuroprotective effect was verified *in vitro*.

The fifth article by Oblak et al. entitled “*Plcg2*^*M*28*L*^ Interacts with High Fat-High Sugar Diet to Accelerate Alzheimer's Disease-relevant Phenotypes in Mice” investigated the susceptibility of late-onset AD (LOAD) in mice with genetic risk factors and a high fat/high sugar diet (HFD). Three mouse models, LOAD1 with *APOE4* and the *Trem2*^*R*47*H*^ variant, LOAD1.*Plcg2*^*M*28*L*^ with the *Plcg2*^*M*28*L*^ variant, and LOAD1.*Mthfr*^677*C*>*T*^ with the *Mthfr*^677*C*>*T*^ variant, were constructed to be fed a HFD or a control diet. The alterations of microglia density, regional brain glucose and perfusion, as well as the transcriptomic changes in LOAD1.*Plcg2*^*M*28*L*^ similar to human AD rather than in LOAD1 and LOAD1.*Mthfr*^677*C*>*T*^ models suggested genotype-specific impact of HFD on developing LOAD.

In the final article, “The Big Picture of Neurodegeneration: A Meta Study to Extract the Essential Evidence on Neurodegenerative Diseases in a Network-Based Approach”, given that dramatically increasing processes involving in neurodegenerative diseases were harder to keep an outline, Ruffini et al. conducted a meta-analysis analyzing four omics layers (genomics, transcriptomics, proteomics, and epigenomics) of four neurodegenerative disorders (AD, PD, HD, and ALS) across 234 studies. Six hallmarks, “cell cycle”, “autophagy/apoptosis”, “ECM/development”, “signal transduction/transport”, “immune system”, and “metabolism”, were categorized to help extracting processes and giving a comprehensive landscape of neurodegeneration to provide a new point of view for the scientific community.

By publishing the mentioned papers, we hope this Research Topic will broaden readers' understanding of potential genetic and epigenetic basis underlying neurodegenerative disease and foster discussion and collaboration.

## Author Contributions

LY and HD drafted the editorial. YG, SW, and HD made the revision. All authors contributed to the article and approved the submitted version.

## Conflict of Interest

The authors declare that the research was conducted in the absence of any commercial or financial relationships that could be construed as a potential conflict of interest.

## Publisher's Note

All claims expressed in this article are solely those of the authors and do not necessarily represent those of their affiliated organizations, or those of the publisher, the editors and the reviewers. Any product that may be evaluated in this article, or claim that may be made by its manufacturer, is not guaranteed or endorsed by the publisher.
